# Archives of Neurology from the Pathological Laboratory of the London County Asylums, Claybury, Essex

**Published:** 1900-03

**Authors:** 


**Archives of Neurology from the Pathological Laboratory of the London County Asylums, Claybury, Essex.** Edited by F. W. MOTT, F.R.S., M.D. Pp. xii., 552. London: P. S. King and Son. [N.D.]

Dr. Collins in his preface calls these archives the first fruits of the establishment of the County Asylums Laboratory at Claybury and the appointment of Dr. F. W. Mott as Superintendent of it. The London County Council are to be congratulated on the result of their enterprise in giving an opportunity for research into the pathology of mental diseases, and most on having enlisted in this work the services of so distinguished a neurologist and pathologist as Dr. Mott. We have no doubt that, good as this volume is, subsequent ones will maintain an equally high level under his superintendence, and will form a most valuable addition to neurological literature.

The present volume deals chiefly with the all-important subject of syphilis in its relation to mental diseases. Dr. Mott has an introductory paper on this subject, and then goes on to describe a number of cases exhibiting the gross lesions of syphilis as it attacks the brain directly. But the more subtle and indirect action of the poison of syphilis on the central nervous system probably gives rise to more cases of disease, and these far less amenable to treatment, than the gross lesions; and in this connection the part played by syphilis in the etiology of general paralysis is of importance. Dr. Mott's views on the relation between syphilis and general paralysis are well known, and he gives here in another paper an account of his observations on the subject. In this connection, another paper by him on twenty-two cases of juvenile general paralysis, deserves a careful perusal. There are several other papers dealing with various important points in the pathology of general paralysis and indeed considerably more than half the book is taken up by the articles, which together constitute a systematic investigation into this disease.

Then follows an important paper on the chemistry of nerve degeneration, and the rest of the volume comprises chiefly reports on the pathology of different cases of interest, all of which will repay study, and would be individually mentioned here if the exigencies of space allowed.

